# DO THE RADIOLOGICAL CRITERIA WITH THE USE OF RISK FACTORS IMPACT THE FORECASTING OF ABDOMINAL NEUROBLASTIC TUMOR RESECTION IN CHILDREN?

**DOI:** 10.1590/0102-6720201700020003

**Published:** 2017

**Authors:** Ana Cláudia Soares PENAZZI, Vivian Siqueira TOSTES, Alexandre Alberto Barros DUARTE, Henrique Manoel LEDERMAN, Eliana Maria Monteiro CARAN, Simone de Campos Vieira ABIB

**Affiliations:** 1 Postgraduate Program in Interdisciplinary Surgical Science; 2 Pediatric Oncology Institute - Support Group for Adolescent and Child with Cancer (IOP-GRAACC) - Federal University of São Paulo - UNIFESP, São Paulo, SP, Brazil.

**Keywords:** Neuroblastoma, Neoplasm staging, Risk factors

## Abstract

**Background:**

The treatment of neuroblastoma is dependent on exquisite staging; is performed postoperatively and is dependent on the surgeon’s expertise. The use of risk factors through imaging on diagnosis appears as predictive of resectability, complications and homogeneity in staging.

**Aim:**

To evaluate the traditional resectability criteria with the risk factors for resectability, through the radiological images, in two moments: on diagnosis and in pre-surgical phase. Were analyzed the resectability, surgical complications and relapse rate.

**Methods:**

Retrospective study of 27 children with abdominal and pelvic neuroblastoma stage 3 and 4, with tomography and/or resonance on the diagnosis and pre-surgical, identifying the presence of risk factors.

**Results:**

The mean age of the children was 2.5 years at diagnosis, where 55.6% were older than 18 months, 51.9% were girls and 66.7% were in stage 4. There was concordance on resectability of the tumor by both methods (INSS and IDRFs) at both moments of the evaluation, at diagnosis (p=0.007) and post-chemotherapy (p=0.019); In this way, all resectable patients by IDRFs in the post-chemotherapy had complete resection, and the unresectable ones, 87.5% incomplete. There was remission in 77.8%, 18.5% relapsed and 33.3% died.

**Conclusions:**

Resectability was similar in both methods at both pre-surgical and preoperative chemotherapy; preoperative chemotherapy increased resectability and decreased number of risk factors, where the presence of at least one IDRF was associated with incomplete resections and surgical complications; relapses were irrelevant.

## RESUMO

**Racional:**

O tratamento do neuroblastoma é dependente de estadiamento primoroso, realizado no pós-cirúrgico e dependente da expertise do cirurgião. O uso de fatores de risco através da imagem ao diagnóstico surge como preditivo de ressecabilidade, complicações e homogeneidade no estadiamento.

**Objetivos:**

Avaliar o critério de ressecabilidade tradicional com os fatores de risco para ressecabilidade, através das imagens radiológicas, em dois momentos no diagnóstico e no pré-cirúrgico analisando a ressecabilidade, complicações cirúrgicas e índice de recidiva**.**

**Métodos:**

Estudo retrospectivo em 27 crianças com neuroblastoma estádios 3 e 4 em abdome e pelve, e com tomografia e/ou ressonância no diagnóstico e pré-cirúrgico, identificando-se a presença de fatores de risco.

**Resultados:**

A idade média das crianças foi de 2,5 anos ao diagnóstico, onde 55,6% estavam acima dos 18 meses, 51,9% eram meninas e 66,7% tinham estádio 4. Houve concordância da ressecabilidade do tumor pelos dois métodos avaliados (INSS e IDRFs) e em ambos os momentos da avaliação, ao diagnóstico (p=0,007) e pós-quimioterapia (p=0,019). Desta forma todos pacientes ressecáveis por IDRFs no pós-quimioterapia tiveram ressecção completa; já nos irressecáveis, 87,5% tiveram ressecção incompleta. Houve remissão em 77,8%, 18,5% recaíram e 33,3% morreram.

**Conclusões:**

A**r**essecabilidade foi semelhante em ambos os métodos tanto no diagnóstico como no pré-cirúrgico. A quimioterapia pré-operatória aumentou a ressecabilidade e diminuição do número de fatores de risco, onde a presença de ao menos um IDRF associou-se às ressecções incompletas e complicações cirúrgicas. As recidivas foram irrelevantes.

## INTRODUCTION

Neuroblastic tumors were described by Wright in 1910 and originate from indiferentiated nervous cells from the neural crest, present at the adrenal medulla, sympathetic ganglia and plexus. For this reason, they can grow in various parts of the body, being 48% adrenal, 25% retroperitoneal, 16% thoracic and are rarer on the neck and pelvis^1^ ^,^ ^2^ ^,^ ^3^ ^,^ ^4^ ^,^ ^11^ ^,^ ^17^. The etiology of neuroblastoma is unknown, but it seems to be related to congenital and genetic anomalies^4^ ^,^ ^7^ ^,^ ^10^. 

Neuroblastoma is the most common extracranial solid tumor in children and represents 10% of childhood cancers (one case for each 7000 children born) and 15% of pediatric cancer deaths^15^ ^,^ ^17^. In São Paulo State, Brazil, it represents 7.7 cases per million children, 30% having until one year of age and 90% aged until 19 months^3^ ^,^ ^4^ ^,^ ^7^ ^,^ ^8^.

They are heterogeneous tumors that can maturate espontaneously or be highly indifferentiated, depending on the biology of the tumor. Thus, biological and molecular factors are related to clinical presentation and prognosis^2^ ^,^ ^3^ ^,^ ^5^ ^,^ ^9^. Signs and symptoms depend on tumor site, but these tumors envolve the main vascular trunks of the body and are often metastatic at diagnosis. Surgical ressection can be very challenging and severe complications can occur, although complete resection is the aim of the surgery. On the other hand, complete resection is often related to favorable histology^7^ ^,^ ^11^ ^,^ ^15^. Clinical symptoms may also be related to catecolamines and VIP producted by the tumor^3^ ^,^ ^4^ ^,^ ^7^ ^,^ ^17^. Image studies are essential for staging and determination of the primary tumor site^2^ ^,^ ^3^ ^,^ ^4^and diagnosis is made through tumor biopsy or bone marrow infiltration of neuroblasts^7^ ^,^ ^10^.

Staging determine risk groups and individual treatment and several systems were proposed. In 1988, INSS *(International*Neuroblastoma Staging System) was presented as a common language for neuroblastoma staging, but it is made postoperatively and dependent on surgical expertise. So, in 2009 the *International*Neuroblastoma Risk Group (INRG) established a new staging system: INRGSS - *International* Neuroblastoma Risk Group Staging System, that evaluates the initial images at diagnosis and describes more than 20 risk factors, the IDRFs -*Image-Defined*Risk Factors*, that predicts surgical risks and challenges for complete resection at diagnosis* ^2^ ^,^ ^6^ ^,^ ^7^ ^,^ ^8^ ^,^ ^13^ ^,^ ^14^ ^,^ ^15^.

The presence of IDRFs is related to surgical complications and incomplete resections and literature uses this in order to equalize the type of resections among different institutions in the world^2^ ^,^ ^5^ ^,^ ^6^ ^,^ ^9^ ^,^ ^13^.

The aim of this study was to compare the traditional resectability criteria (INSS) with the image risk factors criteria (IDRF) for resectability, in two moments: at diagnosis and preoperatively after chemotherapy in a reference institution for pediatric cancer in Brazil.

## METHOD

This is a retrospective review of patients with neuroblastoma treated at the Pediatric Oncology Institute - GRAACC - UNIFESP from 2000 to 2015. Inclusion criteria were: patients with abdominal and pelvic neuroblastomas stages 3 and 4 that had images at diagnosis and before surgery. From 198 patients treated for neuroblastic tumors in the observation period, 64 met the inclusion criteria, but 25 were excluded because images could not be found, nine were referred to the institution after surgery elsewhere and three were initially diagnosed as renal tumors. Thus 27 patients were included in the study and clinical data were collected. Images at diagnosis and post-chemotherapy before surgery were reviewed by surgeons and radiologists. The aim was to evaluate resectability at diagnosis and after chemotherapy based on the presence of IDRFs described by Brisse *et al* (2009) as part of the INRGSS staging system and to determine if the system would impact the surgical decision made for each patient using INSS.

### Statistical analysis

SPSS 20.0 and STATA 12 were used; 5% significance was considered. Kappa and McNemar coeficients were used to compare resectability at diagnosis and post-chemotherapy between INSS and IDRF systems. Uni and multivariate analysis Kaplan-Meier curves and Cox regression models were done.

## RESULTS

Data from 27 children were analyzed. Age varied from 0-9 years, mean 2.5 years, median two years. Mean time from begining of symptoms and diagnosis was 1.4 year; 51.9% were females; 55.6% were aged more than 18 months at disgnosis; 66.7% were stage 4, and it was also verified, similar participations by location of the tumor (p=0,895, Table 1)

**TABLE 1:** Patients characteristics

	n	%
Gender	27	100.0
Female	14	51.9
Male	13	48.1
Age	27	100.0
<12 months	9	33.3
12 - 18 months	3	11.1
> 18 months	15	55.6
Stage	27	100.0
3	9	33.3
4	18	66.7
Site	27	100.0
Right adrenal	8	29.6
Left adrenal	9	33.3
Retroperitoneal	10	37.0
Histology	27	100.0
Unfavorable	3	11.1
Favorable	7	25.9
*Missing	17	63.0
*(diagnosis by bone marrow)		
Initial approach	27	100.0
Chemotherapy	26	96.3
Surgery	1	3.8
Surgery (post-chemotherapy)	26	100.0
No	9	34.6
Yes	17	65.4
Surgical Resection	17	100.0
Complete	10	58.8
Incomplete	7	41.2
Complications	27	100.0
No	16	59.3
Yes	1	3.7
No surgery	10	37.0
Status	27	100.0
Alive without disease	14	51.9
Alive with disease (in treatment)	2	7,4
Alive with disease (bone marrow transplantation)	2	7.4
Deceased	9	33.3
Recurrence	27	100.0
No	22	81.5
Yes	5	18.5

Resectability was compared between INSS and IDRFs at diagnosis (n=27) and after chemotherapy (n=26). One patient was treated with surgery as initial approach and had complete resection (Table 2).

**TABLE 2:** Comparison between INSS and IDRFs

INSS	n	%
Resectable at diagnosis	27	100.0
No	26	96.3
Yes	1	3.7
Resectable after chemotherapy	26	100.0
No	10	38.5
Yes	16	61.5
IDRFs	N	%
Resectable at diagnosis	27	100.0
No	23	85.2
Yes	4	14.8
Resectable after chemotherapy	26	100.0
No	17	65.4
Yes	9	34.6

**FIGURE 1:**
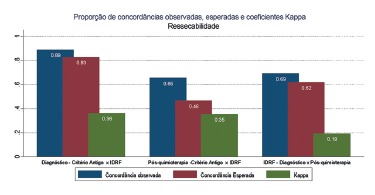
Concordance and Kappa values

As for compared resectability between INSS and IDRFs at diagnosis (Kappa=0,362, p=0,007) and after chemotherapy (Kappa=0,354, p=0,019), fragile but significant agreement results were observed. But when comparing results between diagnosis and post-chemotherapy using IDRFs, no agreement was observed (Kappa=0,194, p=0,107). For the INSS criteria it was not possible to calculate Kappa coefficient because all 26 patients were considered unresectable at diagnosis (Figure 1).

As for the type of surgical resection at diagnosis and post-chemotherapy, for both INSS and IDRFs there was association for the type of resection post-chemotherapy on IDRF(p=0,001), meaning that all patients considered resectable post-chemotherapy by IDRFs had complete resections. On the other hand, 87.5% of patients considered unresectable had incomplete resections (Table 3).

**TABLE 3:** Resectability: INSS/IDRFs, at diagnosis and post-chemotherapy

Resectability	Type of resection				Total		p
	Complete		Incomplete				
INSS	n	%	n	%	n	%	
At diagnosis	10	58.8	7	41.2	17	100.0	1.000
No	9	56.3	7	43.8	16	100.0	
Yes	1	100.0	0	0.0	1	100.0	
Post-cheomtherapy	9	56.3	7	43.8	16	100.0	-
No	-	-	-	-	-	-	
Yes	9	56.3	7	43.8	16	100.0	
IDRFs	n		n		n		p
At diagnosis	10	58.8	7	41.2	17	100.0	0.603
No	7	53.8	6	46.2	13	100.0	
Yes	3	75.0	1	25.0	4	100.0	
Post-chemotherapy	9	56.3	7	43.8	16	100.0	0.001
No	1	12.5	7	87.5	8	100.0	
Yes	8	100.0	0	0.0	8	100.0	

p= Fisher test

**FIGURE 2:**
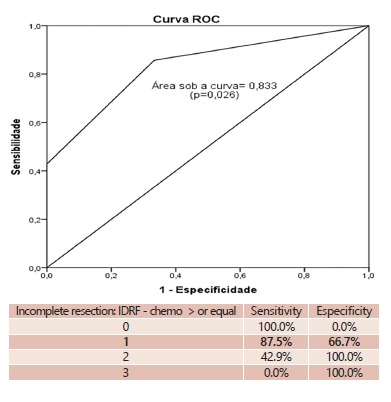
ROC curve for complete resections; IDRFs in incomplete resections

On the ROC curve, a cut point of 1 on the post-chemotherapy IDRF was associated with 87.5% sensitivity and 66.7% of especificity for incomplete resection. Thus, if all post-chemotherapy patients with one or more IDRFs were classified as incomplete resections, 87.5% would be correctly classified and if classified as complete resections, 66.7% would be correctly classified (Figure 2). 

For patients who had surgery after chemotherapy (n=16), differences were observed among number of IDRFs and complete or incomplete resections (p=0,009). The median number of IDRFs was lower for patients that had complete resections.

**FIGURE 3:**
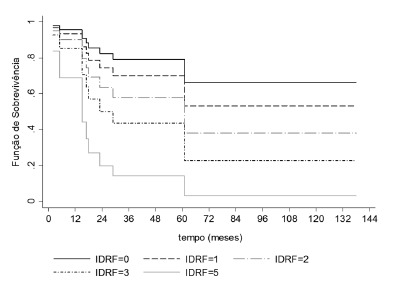
Number of IDRFs and survival

Survival was impacted by the number of IDRFs. The more IDRFs, the worse was the survival (Figure 3).

77.8% (n=18 patients) are alive and there is similar distribution on stages 3 and 4. Four patients are in treatment, 14 are out of treatment: one had complete response and did not have surgery, seven had complete resections, two remain with unresectable tumors after chemotherapy and four had incomplete resections. Nine died, all of them stage 4: eight for progression of disease and one for chemotoxicity. 

## DISCUSSION

Neuroblastoma is a heterogeneous and multifactorial malignancy and its biology impacts survival rates. Multimodal treatment has enhanced the chances of survival and cure^8^. 

Literature shows predominance of males, but in the present series, 51.9% of patients were female (p=0.188). Gender had no influence on survival^5^ ^,^ ^13^ ^,^ ^17^ ^,18^. On the other hand, age at diagnosis of more than 18 months is an independent risk factor for prognosis^5^ ^,^ ^9^. 55.6% were older than 18 months in this series (mean 30 months), showing the prevalence of high staged tumors. 

Concerning site, 62.9% were adrenal, but had no impact on survival (p=0.266).

Surgery is the best initial approach in localized disease, but there is discussion about which is the best to do initially in bigger tumors that encase other structures and advanced staged tumors. The type of surgical resection and staging influence prognosis and some groups advocate complex and risky resections. But others say that aggressive surgery is questionable and has little benefit in high risk patients heavily treated with the multimodal treatment^6^ ^,^ ^17^.

Preoperative chemotherapy is of essence in neuroblastomas that envolve renal vessels, celiac trunk or SMA, after which complete resection possibilities can enhance. Nephrectomies should be prevented, when possible^5^ ^,^ ^8^ ^,^ ^9^.

Mullassery *et al* did a systematic review on the impact of aggressive surgery in stages 3 and 4 neuroblastomas. Complete resections are associated with better prognosis for stage 3, but have limited impact in stage 4 tumors. 

Irtan *et al* compared images from the diagnosis and preoperatively, with the identification of IDRFs in both moments, along with the site and extent of the tumor and the local impact of chemotherapy for surgery. Resectability was enhanced by chemotherapy when using IDRFs: 14.8% at diagnosis and 34.6% after chemotherapy. In our series, post-chemotherapy IDRFs and the type of surgical resection were convergent since patients classified as resectable in the new criteria were actually resected in the past (p=0.001). For those considered unresectable, 87.5% had incomplete resections.

In a previous study at the same institution in 1998, severe surgical complications occurred in 16.4% with 30.7% mortality^1^, but with the advances in chemotherapy, support care and bone marrow transplantation the present series had only one patient with surgical complication and the overall survival that used to be 49.4% is 66.6% today^1^. 

Of 17 patients with high stage disease treated with surgery, only one post-chemotherapy patient had a surgical complication (hemorrhage). This patient had six IDRFs at diagnosis and two IDRFs after chemotherapy, which correlates to challenges in surgery and incomplete resection, which he had. 

The low incidence of surgical complications described, even in high stage disease, can be explained by the fact that the institution is reference for pediatric cancer in Brazil and also the use of preoperative chemotherapy, which reduces the number of IDRFs. 

There have been changes in treatment protocols throughout the years and the relapse rate was 18.5%, lower than related in literature. Survival rates are comparable to those described in the literature (66.6%)^1^ ^,^ ^14^.

There are several limitations in this study; it represents casuistic of a single institution; is retrospective; has a limited number of cases; and the biology of the tumor was not analyzed. Further prospective studies should be conducted to better compare INSS with INRGSS.

## CONCLUSION

Resectability was similar using INSS and IDRFs systems at diagnosis and post-chemotherapy. Chemotherapy enhances the resectability (14.8-34.6%) for the numbers if IDRFs decline. The presence of at least one IDRF was associated with incomplete resections and there was only one surgical complication and low relapse rate. 
